# Deciphering the socio-environmental factors associated with realized heartworm transmission risk in dogs from Portugal and Spain

**DOI:** 10.3389/fvets.2026.1812406

**Published:** 2026-05-22

**Authors:** Rodrigo Morchón, Alfonso Balmori-de la Puente, Elena Infante González-Mohino, Joana Esteves-Guimarães, Pere Busquets, Ana Patrícia Fontes-Sousa, Elena Carretón, José Alberto Montoya-Alonso

**Affiliations:** 1 Zoonotic Diseases and One Health group, Faculty of Pharmacy, Center for Environmental Studies and Rural Dynamization (CEADIR), University of Salamanca, Salamanca, Spain; 2 Biomedical Research Institute of Salamanca (IBSAL), University of Salamanca, Salamanca, Spain; 3 Clínica Veterinária Aanifeira, Santa Maria da Feira, Portugal; 4 Internal Medicine, Faculty of Veterinary Medicine, Research Institute of Biomedical and Health Sciences (IUIBS), University of Las Palmas de Gran Canaria, Las Palmas de Gran Canaria, Spain; 5 Uranovet S.L., Les Franqueses, Barcelona, Spain; 6 RISE-Health, Department of Immuno-Physiology and Pharmacology, Veterinary Hospital of the University of Porto (UPVET), School of Medicine and Biomedical Sciences, University of Porto, Porto, Portugal

**Keywords:** *Dirofilaria immitis*, epidemiology, Portugal, socio-environmental factors, Spain, transmission risk

## Abstract

*Dirofilaria immitis* is a vector-borne zoonotic parasite that is expanding throughout Europe, with the Iberian Peninsula and its archipelagos acting as important endemic areas. Althoug climatic suitability models are available, few studies have analyzed the specific socio-environmental factors associated with Realized Transmission Risk (RTR) in areas with confirmed canine heartworm infection. This study analyzed 3,670 antigen-positive dogs from Spain and Portugal (2023–2024) to characterize these factors. An Ecological Niche Model (ENM) for *Culex pipiens*, integrated with parasite development thresholds, was used to estimate the RTR at the exact geolocation of each infected animal. Multiple linear regression was then applied to assess the association of RTR with geographic, climatic, and host-related variables. The geographic region emerged as the strongest predictor of infection risk, with the insular territories (Balearic and Canary Islands) showing the highest risk coefficients relative to the peninsular baseline. Among climatic classifications, the hot semi-arid climate was significantly associated with increased risk, suggesting that transmission may also become relevant in more arid settings with anthropogenic water sources. Dogs kept indoors also showed a significantly higher risk coefficient than the outdoor/indoor reference category, indicating that indoor housing may noy fully prevent exposure under certain conditions, possibly due to vector endophily and stable domestic microclimates. In addition, RTR was significantly higher in 2024, with marked regional heterogeneity, including a notable increase in northern regions such as Castile and León. Overall, these findings suggest that heartworm transmission is shaped by the interaction of geographic, climatic, and anthropogenic factors rather than occurring randomly. From a One Health perspective, the results support the need for geographically adapted prevention and surveillance strategies, particularly in insular, semi-arid, and emerging inland areas.

## 1 Introduction

Heartworm disease, caused by the nematode *Dirofilaria immitis*, is a vector-borne zoonotic infection whose transmission is intrinsically linked to environmental factors that favor both culicid mosquito vectors and parasite development within the vector (1). Infective third-stage larvae (L_3_) are inoculated into the definitive host during mosquito blood-feeding. Once inside the host, the parasites undergo successive molts until reaching adulthood, localizing mainly in the pulmonary arteries and, in some cases, the right ventricle. This results in progressive vascular and pulmonary damage characterized by proliferative endarteritis, vascular remodeling, oedema, pulmonary hypertension and pulmonary thromboembolism, which may ultimately lead to congestive heart failure and death. The infection affects both domestic and wild canids and felids, with dogs generally regarded as the main reservoir host (2).

In continental Europe, *D. immitis* has shown a progressive expansion toward northern latitudes, driven by a combination of climate change, increased pet movement, inadequate use of chemoprophylaxis and repellents, and limited awareness of the disease, among other factors. Spain and Portugal remain among the areas with the highest endemicity in Europe, and their marked climatic and geographical heterogeneity creates a mosaic of transmission scenarios that warrants detailed assessment of realized transmission risk (3). In both countries, the distribution of *D. immitis* infection in dogs and cats is heterogeneous and is highly influenced by vector abundance and environmental conditions.

Historically, endemic areas in the Iberian Peninsula have included the southern regions, the Mediterranean coast, the Balearic and Canary Islands, and some inland territories. However, epidemiological coverage has not been uniform across the whole peninsula. The emergence of new hotspots with favorable climatic conditions may be related to several interacting factors, including increasing scientific attention to the disease, pet mobility, the expansion of human-modified environments, the presence of stagnant water, and climate change. The current epidemiological landscape therefore reflects a recent and rapid geographic expansion toward central and northern latitudes.

In Portugal, the national prevalence is approximately 5.9%, with higher values in the south reaching 9.4% and peaks of up to 27.3% in central coastal areas (4). In Spain, the northward expansion of the disease is confirmed, and the mean prevalence ranges from 6.25% to 6.47%, with the highest average values reported in the Canary Islands (11.58%) and the Balearic Islands (10.87%) (2, 5). This evolving distribution represents an increasing One Health challenge because of the zoonotic nature of the parasite. In humans, who act as accidental hosts, infection usually manifests as pulmonary dirofilariosis. Although the larvae typically die before reaching adulthood, they may induce benign pulmonary nodules that can be mistaken radiologically for malignant lung tumors (1).

Despite the substantial body of knowledge on the epidemiology of *D. immitis* in Spain and Portugal, few studies have jointly examined additional host and environmental variables—such as age, sex, breed, or habitat—in relation to realized transmission risk (6, 7). Several authors have suggested that host lifestyle, particularly outdoor living, is an important determinant of exposure. Likewise, although age is not necessarily associated with a greater risk of infection per unit of time, prevalence tends to increase with host age, most likely reflecting cumulative exposure over time in endemic areas (4, 5, 8–13).

In these animal populations, heartworm transmission remains a major concern for both veterinary and public health, particularly because infection pressure appears to be increasing in areas traditionally considered to be at low risk. In Spain and Portugal, transmission risk is highly heterogeneous and results from a complex interaction of environmental, climatic, and demographic factors (14). A clear spatial and seasonal pattern has been described, with moderate-to-high risk across large areas of the Iberian Peninsula and the Balearic Islands—especially along the Mediterranean coast and in southern and central regions—whereas colder and higher-altitude areas generally show lower risk. Seasonally, infection risk peaks in summer (July–August) and declines markedly in winter, although low-level transmission potential persists in southern regions, along the Mediterranean coast, and in the Balearic Islands. In the Canary Islands, risk ranges from low to high and remains moderate during winter, suggesting the possibility of year-round transmission, particularly in coastal and low-altitude areas. A similar seasonal pattern has been described in the Azores and Madeira, where the highest risk is concentrated in low-lying and densely populated areas with high vector suitability.

Considering this background, the aim of the present study was to identify the socio-environmental and geographical factors associated with variations in modeled heartworm transmission risk in Spain and Portugal, and to characterize the spatial patterns of transmission in areas with confirmed canine heartworm infection.

## 2 Methods

### 2.1 Location

The study was conducted in the Iberian Peninsula (southern Europe), a territory covering approximately 582,000 km^2^ and comprising mainland Spain and Portugal together with their insular territories. In Spain, the analysis covered the entire national territory, which is administratively organized into 17 autonomous communities and 2 autonomous cities (Ceuta and Melilla), comprising a total of 50 provinces. The Balearic Islands, located in the Mediterranean Sea, and the Canary Islands, located in the Atlantic Ocean off the northwestern coast of Africa, were specifically included. In Portugal, the study area comprised the mainland territory, which is divided into 18 districts, as well as the two autonomous island regions of the Azores and Madeira, both located in the Atlantic Ocean (15, 16).

From a climatic perspective, the hot-summer Mediterranean climate (Csa) pre-dominates across much of the Iberian Peninsula and the Balearic Islands. This climate is characterized by average temperatures exceeding 22 °C in the warmest month and is pre-dominant in the southern half of the peninsula, along the Mediterranean coast, and throughout the Balearic archipelago. In contrast, the northern coast and areas near the Pyrenees are dominated by a temperate oceanic climate (Cfb), characterized by mild summers (mean temperature of the warmest month < 22 °C) and at least four months with average temperatures above 10 °C. In the northeastern Iberian Peninsula, both Cfb and humid subtropical (Cfa) climates are present, the latter lacking a defined dry season. Toward the northwest, the warm-summer Mediterranean climate (Csb) pre-dominates, with all months showing average temperatures below 22 °C. In southeastern regions, the cold semi-arid climate (BSk) prevails, characterized by low precipitation, and coexists with areas of hot semi-arid (BSh) and hot desert (BWh) climates, where mean annual temperatures exceed 18 °C.

Regarding the Atlantic insular territories, the Canary Islands show marked climatic variability. The eastern islands are dominated by a desert climate (BWh), whereas the western islands combine temperate climates (Csb, Csa) in the north with arid climates (BWh and BSh) in the south. The Azores and Madeira archipelagos display pre-dominantly oceanic and temperate conditions. In Madeira, the prevailing climate is temperate (Csb), although dry conditions are observed across much of Porto Santo Island (Bsh and hot steppe climate). In the Azores, the pre-dominant climate is temperate with no dry season and a mild summer (Cfb), although other subtypes occur locally on some islands (17, 18).

### 2.2 Samples

The study included 3,670 domestic dogs that tested positive for the detection of *D. immitis* antigens using a commercial immunochromatographic assay (Uranotest^®^ Dirofilaria, Uranovet, Barcelona, Spain). Testing was performed in veterinary clinics and hospitals in Spain and Portugal between January 2023 and December 2024. According to the manufacturer, the test has a sensitivity of 94% and a specificity of 100% compared with necropsy.

Participation by veterinary clinics and dog owners was voluntary. Dogs were eligible for inclusion if they were older than 6 months of age. Epidemiological data were also recorded, including year of diagnosis, age, sex, habitat, breed, location, climate, autonomous community/district (Spain/Portugal), region, and postcode.

### 2.3 Risk assessment of *Dirofilaria immitis* infection

To assess the risk of *D. immitis* infection across the Iberian Peninsula, the Balearic Islands, the Azores, Madeira, and the Canary Islands, the methodology described by Infante González-Mohino et al. (14) was applied. First, habitat suitability models (Ecological Niche Models, ENMs) for *Cx. pipiens* were developed for each region using vector geolocation data and a set of bioclimatic and environmental variables obtained from published studies and public repositories (14).

Vector occurrence points were processed using QGIS software version 3.34.15 (19), together with 19 bioclimatic variables related to temperature and precipitation for the period 1970–2000 (20), as well as the following hydrological layers: rivers, lakes, lagoons, irrigated croplands, and artificial and natural stagnant water bodies (14). These layers were included because they represent water sources relevant to the transmission dynamics of organisms involved in the cycle, particularly the vector. The ENMs were generated using the KUENM package in R (version 1.1.10). Variables were selected after multicollinearity analysis based on Pearson's correlation coefficient (r < 0.8), performed in R software version 4.4.2 (21).

Second, a map of the potential number of *D. immitis* generations was generated in R version 4.3.0, taking into account the minimum temperature threshold below which larval development cannot occur within the vector. Finally, the habitat suitability models for *Cx. pipiens* and the map of potential *D. immitis* generations were combined with equal weighting (50:50) using the raster calculator in ArcMap 10.8 (22).

### 2.4 Statistical analysis

Statistical analyses were performed using R software version 4.2.3 (21). First, a descriptive analysis was conducted to summarize the number of positive cases and the corresponding percentages for each epidemiological category included in the study.

A two-stage analytical framework was then applied. In the first stage, the Ecological Niche Model (ENM) was used to estimate transmission risk based on macro-environmental, climatic, and hydrological variables. In the second stage, multiple linear regression was performed using the continuous ENM output as the dependent variable, defined here as Realized Transmission Risk (RTR). RTR values were extracted at the exact geographical coordinates of each positive dog record. Because the analysis was restricted exclusively to confirmed positive cases of dirofilariosis, it specifically focused on areas with demonstrated effective transmission.

The RTR was evaluated against a set of predictor variables, including host factors (breed, sex, and age), geographic factors (autonomous community/district and region), environmental factors (Köppen climate classification and habitat type), and temporal factors (year of diagnosis). Multiple linear regression was used to assess the association between the local socio-environmental context and the ENM-predicted risk at these confirmed transmission foci. After verifying acceptable model diagnostics, including multicollinearity thresholds (GVIF∧(1/(2*Df)) < 2) and residual assumptions, a bidirectional stepwise variable selection procedure based on the Akaike Information Criterion (AIC) was applied to identify the most relevant variables. In the final model, lower-risk factor levels were relevelled as the reference categories in order to estimate the measurable increase in transmission risk relative to the lowest-risk scenario.

Finally, *post hoc* pairwise comparisons for significant categorical variables retained in the final model were performed using Fisher's exact test, which is appropriate for datasets with small expected frequencies in contingency tables. Bonferroni correction was applied for multiple comparisons using the pairwise Fisher test function in R. Statistical significance was set at p < 0.05.

## 3 Results

### 3.1 Geolocation of infected animals on the risk map

**Figure 1:**
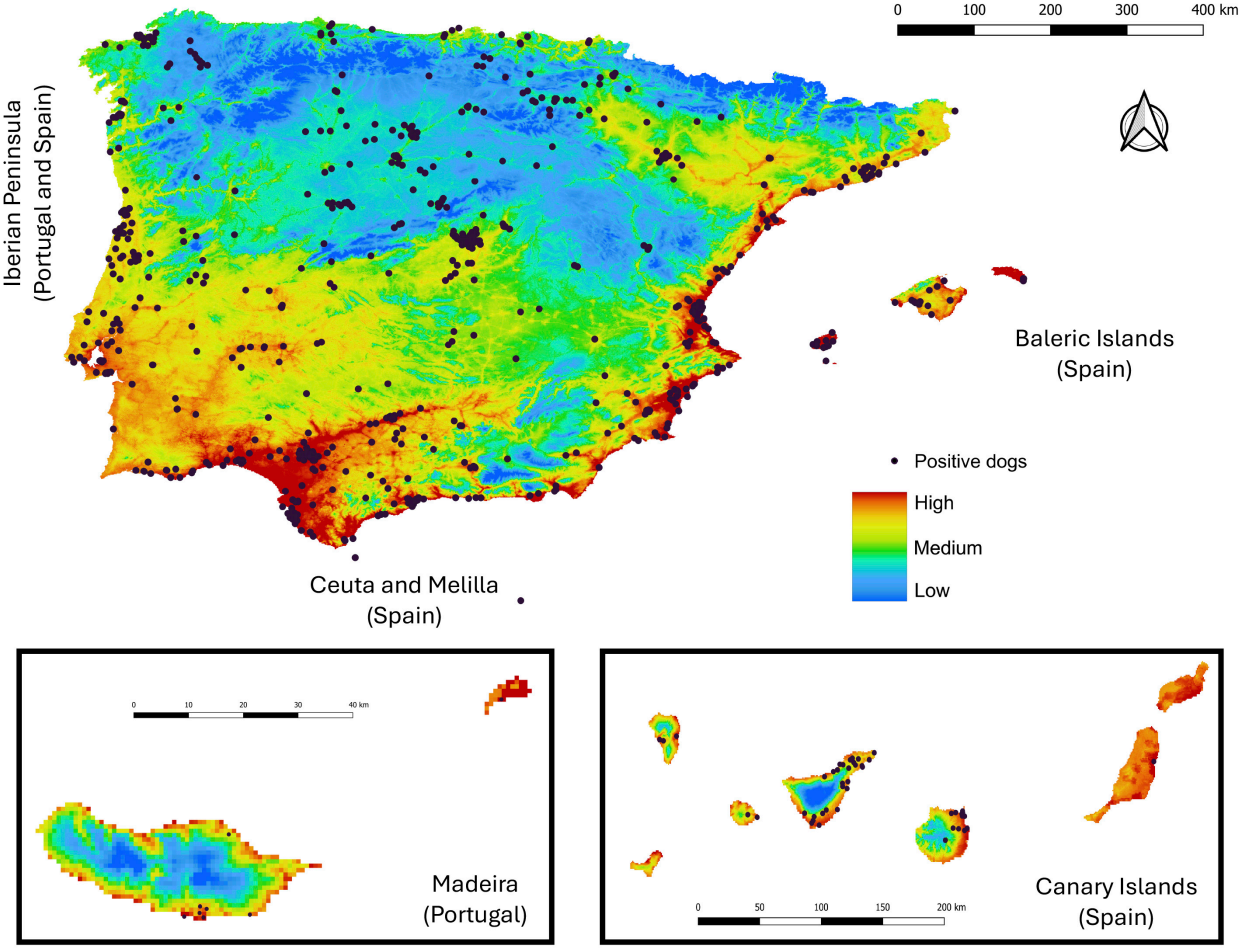
Spatial distribution of *Dirofilaria immitis* transmission risk in Spain and Portugal. The base map illustrates the environmental suitability gradient predicted by the Ecological Niche Model, with the color scale representing risk level: blue (low), green/yellow (medium), and red (high). Black dots indicate the exact georeferenced locations of *D. immitis*-positive dogs included in the study. Enlarged panels are shown for the island territories (Balearic Islands, Canary Islands, and Madeira) and the autonomous cities of Ceuta and Melilla.

The spatial projection of *D. immitis* transmission risk in Spain and Portugal, together with the geolocation of the 3,670 positive dogs (Figure 1), revealed marked geographical heterogeneity across the Iberian Peninsula and its archipelagos. A correspondence was observed between areas of high predicted suitability and the concentration of diagnosed positive cases.

In the Iberian Peninsula, positive dogs located in very high-risk (24.4%) and high-risk (48.3%) areas were distributed mainly along the Mediterranean coast and the southern Atlantic coast, extending inland through the basins of the major river systems. In contrast, high-altitude regions and the central plateau were characterized pre-dominantly by medium-risk (27%) and low-risk (0.3%) conditions.

In the extra-peninsular territories, both the Canary Islands and the Balearic Islands showed pre-dominantly high-risk values, in agreement with the high density of positive cases observed. In the Canary Islands, marked intra-island variability was identified: positive dogs in very high-risk areas (71.7%) were mainly located in low-lying coastal zones, whereas dogs from higher-altitude and mid-altitude central areas were distributed in medium-risk (24.5%) and high-risk (3.8%) zones. In the Balearic Islands, 65.4% of positive dogs were located in very high-risk areas and 34.6% in high-risk areas, with no cases recorded in medium-, low-, or very low-risk zones. In Madeira, the risk pattern was concentrated mainly along the coastal perimeter, where positive cases clustered in very high-risk (77.8%) and high-risk (22.2%) areas, with no cases detected in medium-, low-, or very low-risk zones. No positive cases were reported in the Azores. Finally, in the autonomous cities of Ceuta and Melilla, infected dogs were found exclusively in high-risk areas along the North African coast.

### 3.2 Epidemiological profile of infected dogs

Of the dogs included in the study, 45.42% (1,667/3,670) were diagnosed in 2023 and 54.58% (2,003/3,670) in 2024. Regarding sex, 54.58% (2,003/3,670) were male and 45.42% (1,667/3,670) were female. Age was classified into five groups: < 1 year (1.96%; 72/3,670), 1–4 years (19.73%; 724/3,670), 5–10 years (63.05%; 2,314/3,670), 11–15 years (14.03%; 515/3,670), and >15 years (1.23%; 45/3,670).

Regarding habitat, 11.99% (440/3,670) of the dogs were kept indoors (always inside the house), 54.31% (1,993/3,670) were kept outdoors (always outside), and 33.71% (1,237/3,670) were classified as indoor/outdoor, spending between 1% and 50% of their time outdoors.

With respect to climate, 9.32% (342/3,670) of the dogs were located in BSh areas, 3.71% (136/3,670) in BSk areas, 0.84% (31/3,670) in BWh areas, 4.11% (151/3,670) in Cfb areas, 68.20% (2,503/3,670) in Csa areas, and 13.81% (507/3,670) in Csb areas.

A total of 86 breeds were recorded among the infected dogs (Supplementary Table S1), of which 21 breeds (21/86) included more than 40 infected animals. Mixed-breed dogs accounted for 37.06% (1,360/3,670) of cases, followed by Labrador Retrievers (4.99%; 183/3,670), Yorkshire Terriers (4.20%; 154/3,670), Golden Retrievers (3.98%; 146/3,670), Cocker Spaniels (3.98%; 143/3,670), Boxers (3.81%; 140/3,670), German Shepherds (3.22%; 118/3,670), Poodles (2.94%; 108/3,670), French Bulldogs (2.64%; 97/3,670), Podencos (2.56%; 94/3,670), Spanish Greyhounds (2.34%; 86/3,670), Chihuahuas (2.15%; 79/3,670), Maltese dogs (1.72%; 63/3,670), Beagles (1.53%; 56/3,670), German Pointers (1.50%; 55/3,670), Fox Terriers (1.44%; 53/3,670), Schnauzers (1.42%; 52/3,670), Mastiffs (1.39%; 51/3,670), Brittany Spaniels (1.23%; 45/3,670), American Staffordshire Terriers (1.20%; 44/3,670), and Dachshunds (1.17%; 43/3,670). The remaining breeds (65/86) each accounted for less than 1% of infected animals.

Regarding geographic distribution in Spain (Supplementary Table S2), which accounted for 83.38% of all cases, 28.34% (1,040/3,670) were recorded in Andalusia, 11.77% (432/3,670) in Catalonia, 6.87% (252/3,670) in the Canary Islands, 6.78% (249/3,670) in Madrid, 6.46% (237/3,670) in the Valencian Community, 3.92% (144/3,670) in Extremadura, 3.71% (136/3,670) in Castile and León, 3.24% (119/3,670) in Aragon, 2.89% (106/3,670) in the Balearic Islands, 2.34% (86/3,670) in Galicia, 2.29% (84/3,670) in Murcia, and 1.63% (60/3,670) in Castilla-La Mancha. The remaining autonomous communities (Asturias, La Rioja, Basque Country, Cantabria, and Navarre), together with the autonomous cities of Ceuta and Melilla, each accounted for fewer than 40 infected dogs (< 1% of the total). In Portugal, which represented 16.62% of all cases, 6.51% (239/3,670) were recorded in Centro, 3.43% (126/3,670) in Algarve, 2.32% (85/3,670) in Setúbal, 1.23% (45/3,670) in Oeste e Vale do Tejo, and 1.04% (38/3,670) in Alentejo. Lisbon, Madeira, and Norte each accounted for fewer than 40 infected dogs (< 1% of the total).

### 3.3 Multiple linear regression

The final regression model identified factors that significantly explained the spatial variability of the ENM-predicted transmission risk within the documented transmission foci (R^2^ = 0.75).


$$\begin{array}{l} \text{Y }=\text{ Incercept}+\text{ Σ β Autonomous Community}/\text{Region}\\ \;+\text{ Σ β Climate}+\text{Σ β Habitat }+\text{Σ β Year}+\epsilon \end{array}$$

The model Intercept represented the baseline RTR, corresponding to the average ENM-predicted risk score for a canine dirofilariosis case located in the lowest-risk reference region (Castile and León), under the reference climate conditions (Cfb), within the reference habitat category (outdoor/indoor), and in the reference year (2023). Accordingly, all positive coefficients indicate an increase in the ENM risk score relative to this low-risk baseline.

**Figure 2:**
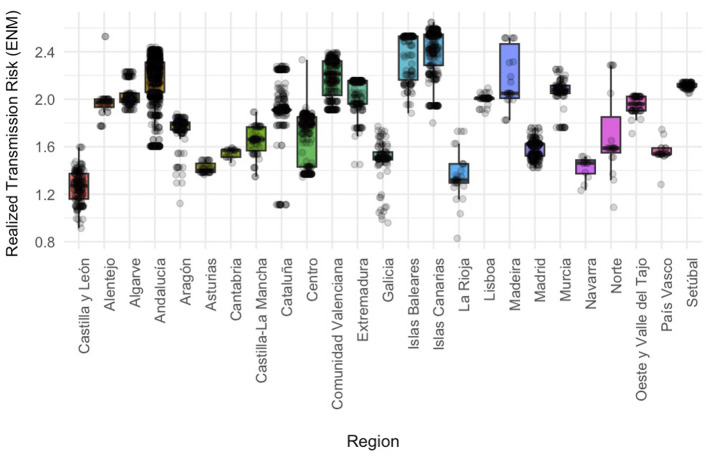
Relationship between autonomous community/region and transmission risk. Boxplots comparing regional categories based on the georeferenced locations of positive canine cases (shown as individual points), with Realized Transmission Risk (RTR) scores estimated from an Ecological Niche Model of *Cx. pipiens* weighted by the potential number of *D. immitis* developmental generations. Whiskers extend to 1.5 times the interquartile range, and the box represents the 50% of observations around the median.

The autonomous community/region variable showed the largest marginal effect on predicted risk (p < 0.05 for all listed regions). As expected from the low-risk baseline strategy, all regional coefficients were positive, indicating a higher RTR in every listed region compared with the reference region (Castile and León). The largest increases in risk were observed in the insular territories of the Balearic Islands (1.1134) and the Canary Islands (1.0814). Conversely, La Rioja (0.1114), Navarra (0.1739) and Asturias (0.1794) showed the smallest, although still significant, increases in risk relative to the baseline (Figure 2, Supplementary Table S3).

**Figure 3:**
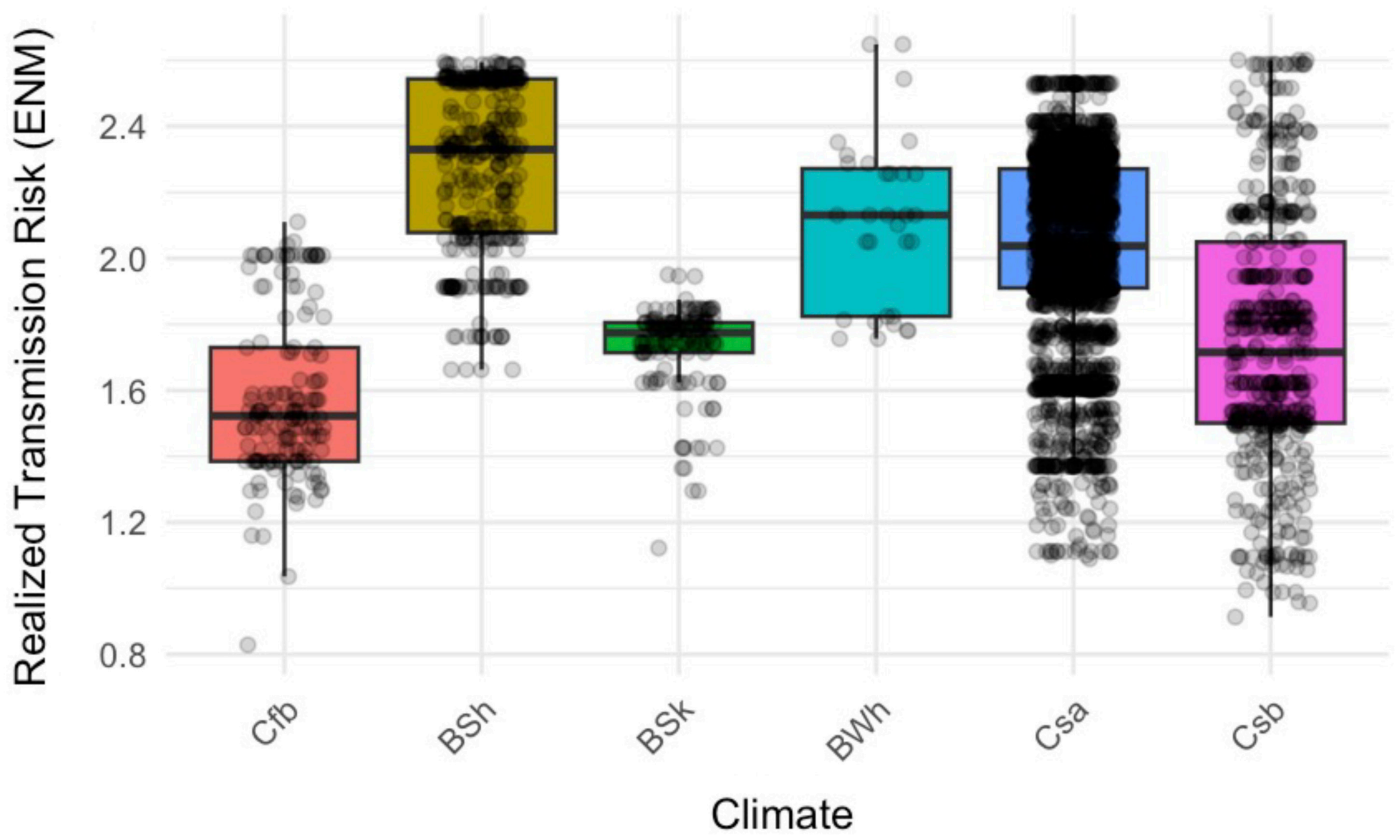
Relationship between Köppen-Geiger climate classification and transmission risk. Boxplots showing Realized Transmission Risk (RTR) values for each climate category based on the georeferenced locations of positive canine cases, with individual observations displayed as points. RTR was estimated using an Ecological Niche Model of *Cx. pipiens* weighted by the potential number of *D. immitis* developmental generations. Boxes represent the interquartile range, the median is shown as a horizontal line, and whiskers extend to 1.5 times the interquartile range.

Among the Köppen climate classifications included in the model, only the BSh category was significantly associated with RTR (p < 0.01). Holding the remaining variables constant, this climate type was associated with a 0.1203-unit increase in predicted risk relative to the reference climate category (Cfb), which represented the lowest-risk climatic scenario among the sampled case locations. The remaining climate types, including Csa and Csb, did not differ significantly from the baseline climate category (Figure 3, Supplementary Table S3).

**Figure 4:**
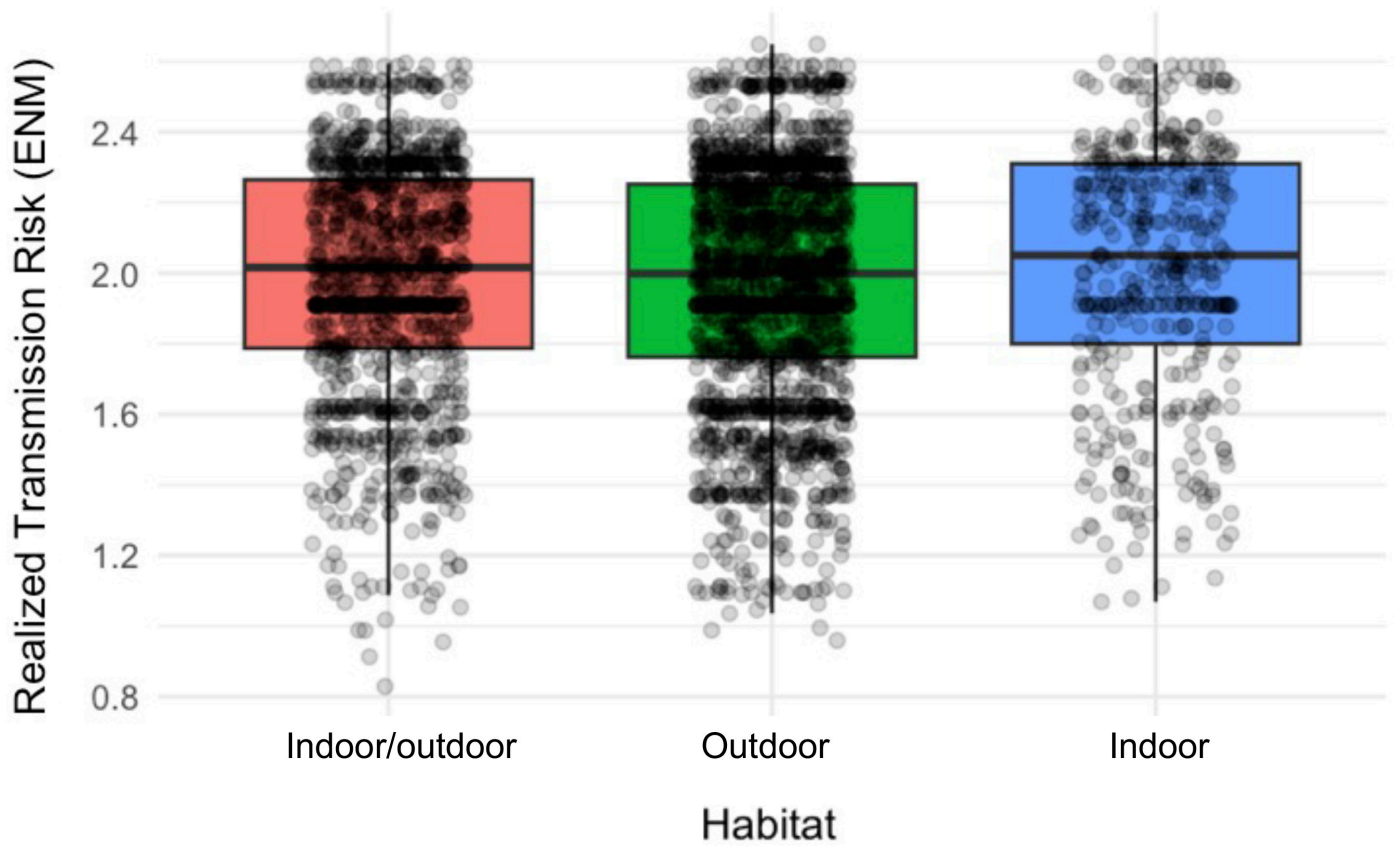
Relationship between habitat type and transmission risk. Boxplots showing Realized Transmission Risk (RTR) values for each habitat category based on the georeferenced locations of positive canine cases, with individual observations displayed as points. RTR was estimated using an Ecological Niche Model of *Cx. pipiens* weighted by the potential number of *D. immitis* developmental generations. Boxes represent the interquartile range, the median is shown as a horizontal line, and whiskers extend to 1.5 times the interquartile range.

Habitat type was also significantly associated with RTR. The indoor category showed a significant positive effect (coefficient = 0.0334; p < 0.001), whereas the outdoor category showed only a small positive coefficient (0.0029). Compared with the reference category (outdoor/indoor), the indoor habitat was associated with a 0.0334-unit increase in RTR, while the effect observed for the outdoor habitat was minimal, indicating a risk profile close to that of the baseline category (Figure 4, Supplementary Table S3).

**Figure 5:**
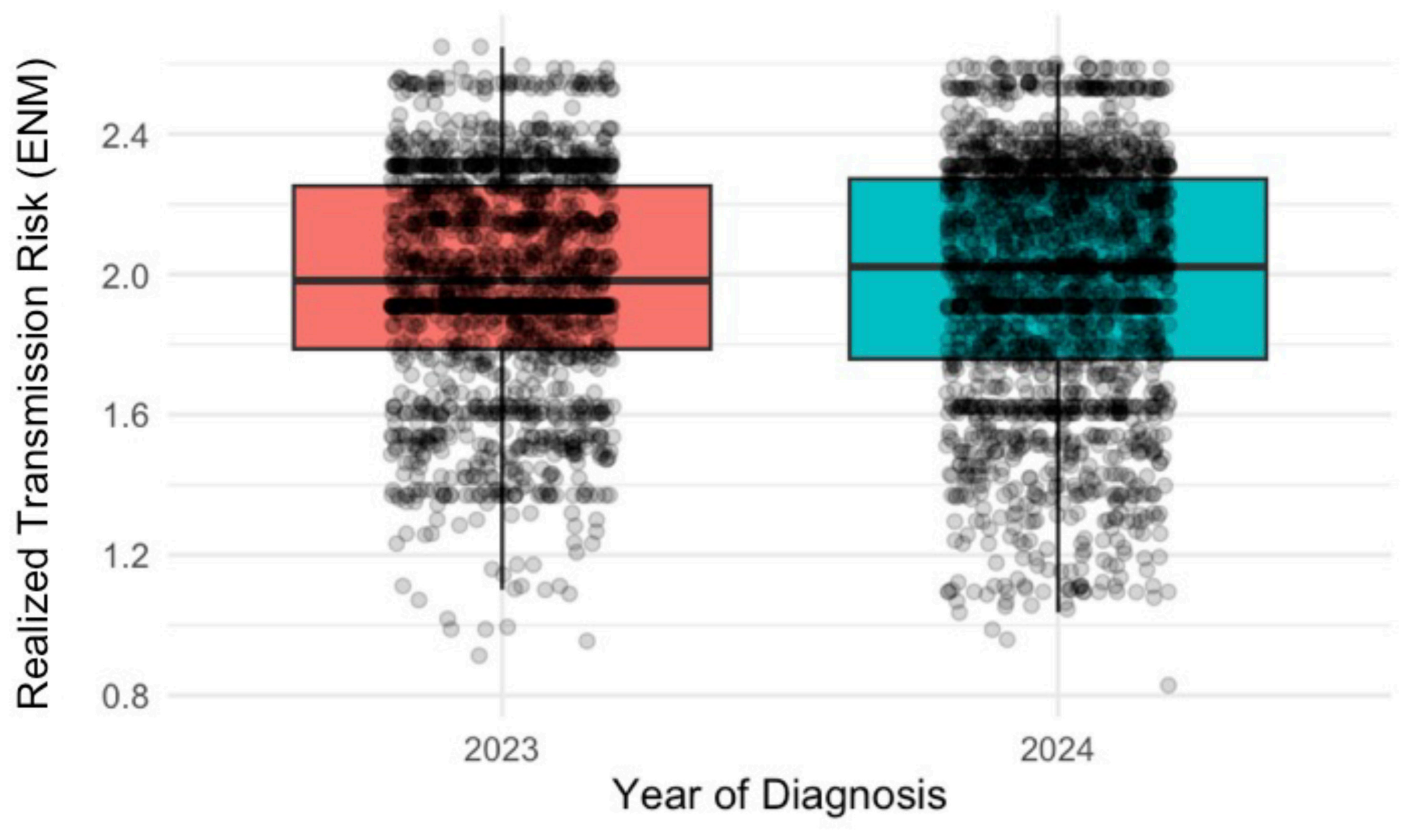
Relationship between diagnosis year and transmission risk. Boxplots showing Realized Transmission Risk (RTR) values for each diagnosis year based on the georeferenced locations of positive canine cases, with individual observations displayed as points. RTR was estimated using an Ecological Niche Model of *Cx. pipiens* weighted by the potential number of *D. immitis* developmental generations. Boxes represent the interquartile range, the median is shown as a horizontal line, and whiskers extend to 1.5 times the interquartile range.

Finally, year of diagnosis was retained in the final model, with 2024 showing a significant positive effect (coefficient = 0.0204; p < 0.001). This indicates that, on average, cases diagnosed in 2024 were associated with slightly higher RTR values than those diagnosed in 2023 (Figure 5, Supplementary Table S3).

### 3.4 Association between categorical relevant variables

Pairwise comparisons between significant categorical variables were performed using Fisher's exact test. Significant associations were identified for the following variable pairs: autonomous community–habitat, region–climate, autonomous community–year of diagnosis, and climate–year of diagnosis (p < 0.001 in all cases).

**Figure 6:**
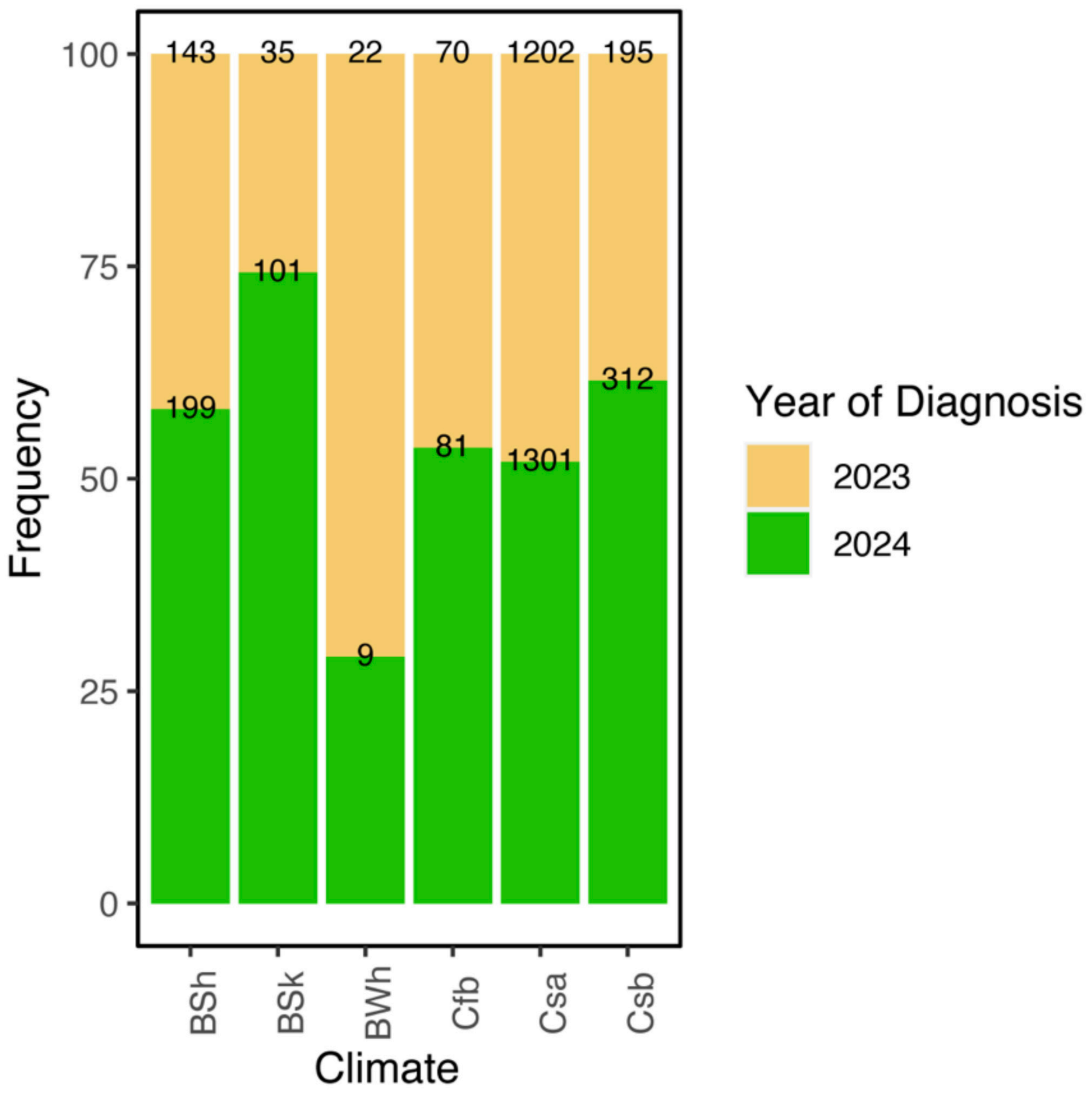
Association between Köppen-Geiger climate classification and diagnosis year. Stacked bar plot showing the distribution of climate categories across the two sampled years (2023 and 2024). Numerical labels within each segment indicate the number of infected dogs (*n*).

The association between Köppen climate classification and year of diagnosis showed that the temporal distribution of cases differed across climate categories. Although the Csa climate included the largest overall number of cases, the BSk category showed the strongest temporal skew, with approximately 74% of cases recorded in 2024. By contrast, the BWh category was more strongly represented in 2023. These findings indicate that the increase in cases observed in 2024 was not uniform across all climate types (Figure 6, Supplementary Table S4).

**Figure 7:**
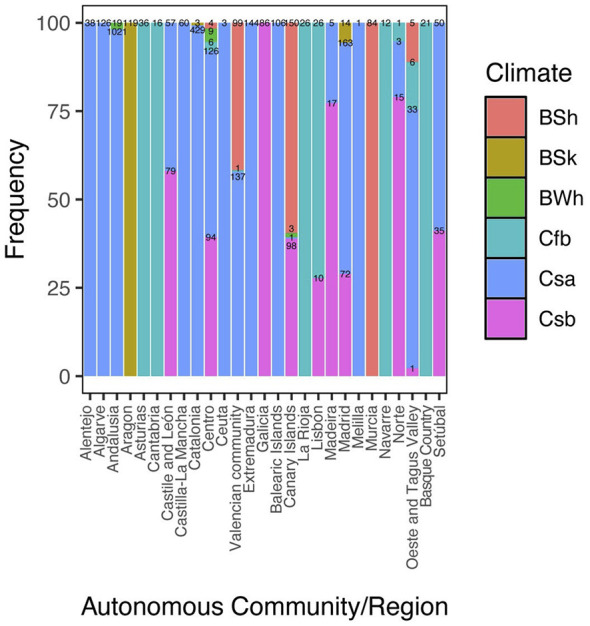
Association between autonomous community/region and Köppen-Geiger climate classification. Stacked bar plot showing the distribution of cases across regions and climate categories. Numerical labels within each segment indicate the number of infected dogs (*n*).

The association between region and climate classification was also significant, indicating a structured distribution of climate categories across the study area. Certain regions were pre-dominantly associated with specific Köppen climate types; for example, the Canary Islands were mainly represented by BSh and Csb climates. These results show substantial overlap between both variables in the dataset (Figure 7, Supplementary Table S4).

**Figure 8:**
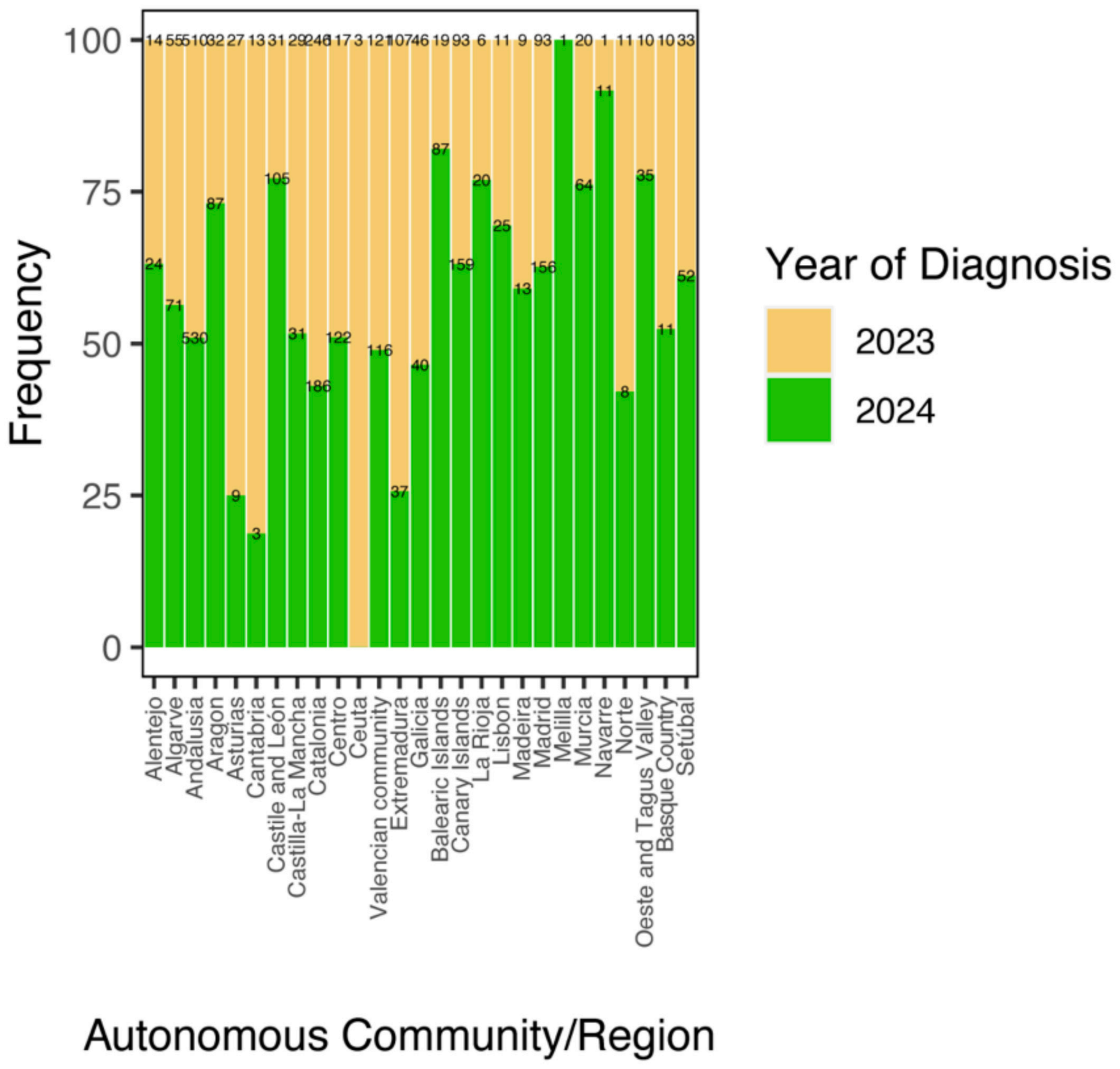
Association between autonomous community/region and diagnosis year. Stacked bar plot showing the distribution of cases across regions in 2023 and 2024. Numerical labels within each segment indicate the number of infected dogs (*n*).

A significant association was likewise observed between autonomous community and year of diagnosis, indicating that the temporal increase in cases varied among regions. Some autonomous communities showed a stronger concentration of cases in 2024, whereas others displayed a more even distribution between the two study years. For example, in Castile and León, approximately 75% of the total cases were recorded in 2024, whereas in Extremadura only about 25% of cases were recorded in 2024, with most cases occurring in 2023 (Figure 8, Supplementary Table S4).

**Figure 9:**
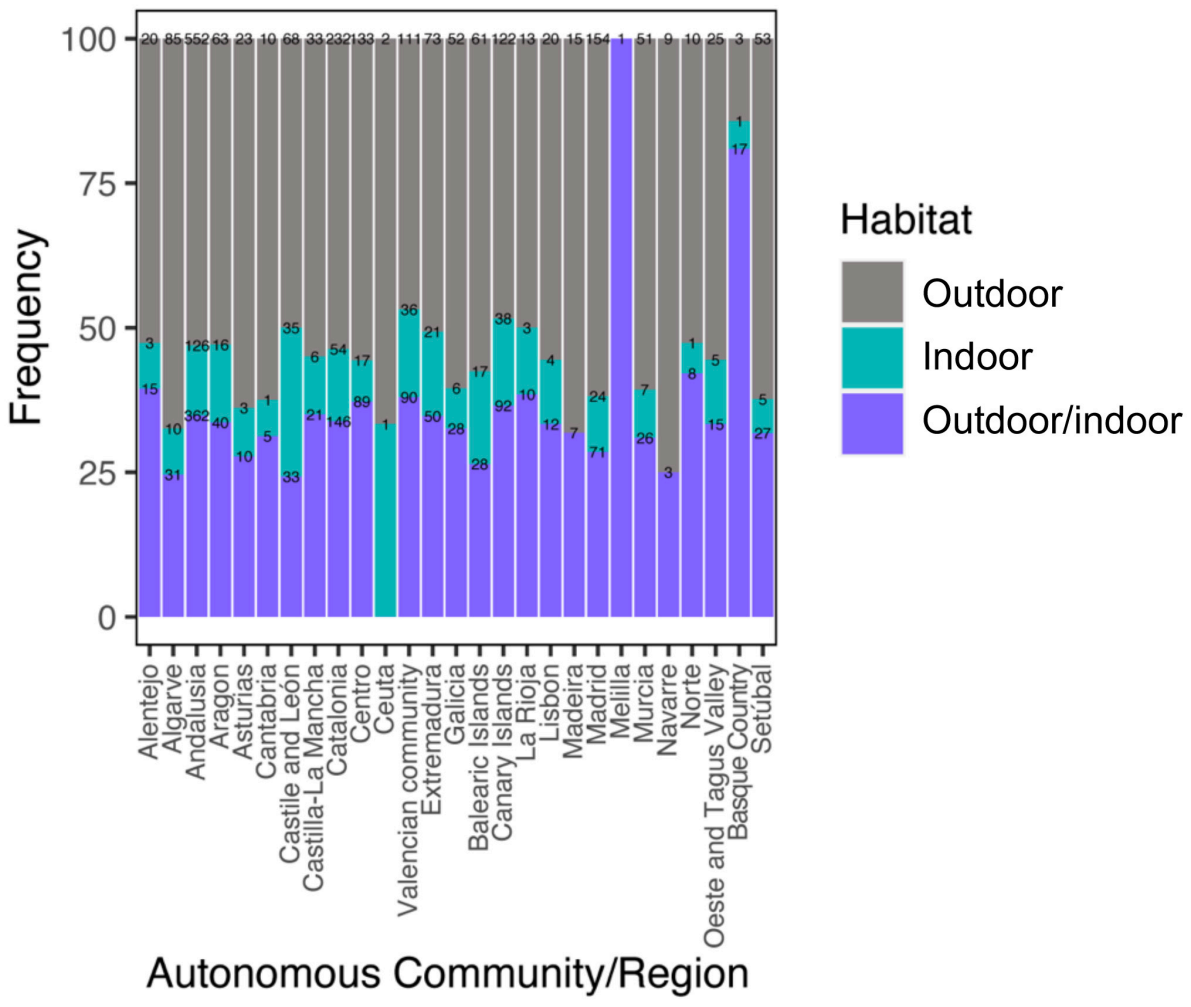
Association between autonomous community/region and habitat type. Stacked bar plot showing the distribution of cases across regions and habitat categories (outdoor, indoor, outdoor/indoor). Numerical labels within each segment indicate the number of infected dogs (*n*).

Finally, the association between autonomous community and habitat revealed regional differences in the environments in which infected dogs were recorded. While outdoor habitat pre-dominated in some autonomous communities, others showed a more mixed distribution across habitat categories: Andalucía, the autonomous community with the highest case load, concentrated the largest number of cases in the outdoor habitat (*n* = 552). By contrast, some regions with a lower total number of cases showed a relatively greater proportional distribution in other habitat categories; for instance, País Vasco showed a higher representation in the outdoor/indoor category. Supplementary Table S4 summarizes all significant pairwise associations (Figure 9).

## 4 Discussion

The present study provides a comprehensive and exhaustive epidemiological and geospatial assessment of heartworm disease in Southern Europe. By integrating data from 3,670 dogs infected with *D. immitis* with ENMs for the main vector species in Spain and Portugal, together with information on parasite development within the vector, we characterized not only the distribution of infection but also the socio-environmental factors associated with variation in RTR across the analyzed regions. Overall, the final regression model suggests that *D. immitis* transmission is shaped by a predictable interaction of geographical, climatic, and anthropogenic variables rather than occurring randomly.

The general epidemiological profile identified in this study, characterized by middle-aged, mixed-breed, outdoor dogs from Mediterranean climate zones, is consistent with previous descriptions in the veterinary literature (4, 5, 12, 23). However, the multivariate analysis also revealed important spatial nuances. The category “Region” emerged as the strongest predictor of risk, with the insular territories (the Balearic and Canary Islands) showing the highest risk coefficients compared with mainland Spanish autonomous communities such as Castile and León. This finding supports the classification of both archipelagos as highly endemic areas. In the Canary Islands, the elevated infection risk, together with the number and distribution of infected animals, may be related to the particular geomorphological and climatic conditions of the archipelago, which can favor year-round transmission (8, 24). The observed intra-island variability, with higher risk in low-lying coastal areas than in high-altitude zones, is also consistent with the ecological requirements of culicid vectors, such as *Cx. theileri* and *Cx. pipiens*, which are associated with stable temperatures and humid microclimates, often linked to human settlements and tourist areas. Similarly, in the Balearic Islands, insularity, host density, and the persistence of wetland environments may contribute to sustained transmission, in agreement with recent seroprevalence studies from the area (3, 9, 25).

In the Iberian Peninsula, the north–south and coast–inland risk gradients generally followed the expected bioclimatic patterns. However, the detection of positive cases in areas classified by our model as medium- or low-risk, mainly in the central plateau and northern regions, suggests the presence of localized transmission hotspots that may not be captured at the macroclimatic scale. Although broad hydrological variables were not retained as primary predictors in the final model, local microenvironmental conditions may still facilitate transmission. For example, urban heat islands may provide the thermal conditions required for *D. immitis* to complete larval development to the infective L_3_ stage, even in regions where the general climate would otherwise be less favorable, provided that suitable small-scale mosquito breeding sites are also present (6).

The strong association observed between region and Köppen climate classification also deserves consideration from a modeling perspective. In the study area, some regions are pre-dominantly represented by specific climate types, which generates partial overlap between both predictors. Consequently, the regional variable may capture not only administrative or geographic differences, but also part of the localized climatic signal associated with vector suitability. This should be taken into account when interpreting the comparatively large marginal effect of region in the final model, as part of that effect may reflect the underlying bioclimatic structure of the territory rather than an exclusively regional effect.

One notable finding was the association between the BSh climate (hot semi-arid) and increased transmission risk, as it was the only Köppen classification that remained significant relative to the reference category. Although it may seem counterintuitive that a climate with limited rainfall could favor a mosquito-borne parasite, BSh areas combine high temperatures, which can accelerate L_3_ larval development within the vector, with the presence of anthropogenic water sources such as irrigation systems, swimming pools, and residential developments.

The lack of statistical significance for Mediterranean climates (Csa/Csb) should not be interpreted as an absence of risk, but rather as indicating that the transmission risk in these areas may already be captured by the regional variable in the model. In contrast, the BSh climate appears to stand out from the other climate categories, suggesting that heartworm transmission may also become relevant in more arid settings. This pattern could be related to the adaptation of invasive vectors such as *Ae. albopictus* or the persistence of *Cx. pipiens* in peri-urban aquatic environments (26). This interpretation is also consistent with climate change projections for southern Europe, which predict increasing aridification and may favor the concentration of transmission in specific ecological niches where high temperatures and artificial water sources coexist (3).

The significance of the year 2024 and the BSh climate in our model suggests that unusual climatic conditions during the sampling period, such as the exceptional heatwaves recorded in the Iberian Peninsula in 2023 and 2024, may have contributed to increased transmission. These thermal anomalies may have accelerated the extrinsic development of *D. immitis* larvae within the vector and, in semi-arid environments such as BSh areas, may also have promoted greater contact between vectors and hosts around anthropogenic water sources, including irrigation systems and urban parks. Together, these factors may help explain why transmission risk in these settings appears to exceed what would be expected on the basis of broader macroclimatic patterns alone, particularly when compared with more temperate Mediterranean climates such as Csa and Csb.

The temporal increase observed in 2024 was not homogeneous across all autonomous communities. Some regions, such as Castile and León, showed a marked concentration of cases in the most recent year, a pattern that may reflect either recent geographic expansion of transmission or increased surveillance and diagnostic activity in previously under-recognized areas. In contrast, regions such as Extremadura showed a more stable temporal distribution, with a greater proportion of cases already recorded in 2023, which may be more compatible with a previously established endemic situation. These regional differences suggest that the recent increase in detected cases does not respond to a single epidemiological process, but rather to a combination of emerging foci, local ecological suitability, and variable diagnostic intensity across the territory.

Regarding habitat, the model indicated that the indoor category was significantly associated with a higher RTR than the reference category (outdoor/indoor), with a coefficient slightly above that observed for the outdoor category. Although outdoor dogs accounted for most detected cases, the model suggests that the intensity of transmission risk may be greater in indoor environments under certain conditions. This finding challenges the assumption that indoor housing alone provides effective protection against heartworm infection. Endophilic mosquito behavior may facilitate exposure inside dwellings, particularly in urban settings, and a lower perceived risk may reduce the use of chemoprophylaxis or repellents by owners (4, 5). Moreover, stable indoor microclimates may support adult mosquito survival during colder periods and thereby prolong the seasonal window for transmission.

The significant association between autonomous community and habitat type indicates that the environmental context of detected cases is not homogeneous across the study area. In highly endemic regions such as Andalucía, the pre-dominance of cases recorded in outdoor habitats suggests that outdoor transmission settings remain epidemiologically important, likely reflecting the combined effect of favorable vector ecology and the management of dogs with greater exposure to mosquito bites. By contrast, in regions with a lower overall case burden, the relatively greater proportional representation of mixed or non-exclusively outdoor habitats may indicate a more relevant contribution of domestic or peri-domestic transmission settings in these localized foci. This pattern should be interpreted cautiously, as habitat distribution may also be influenced by owner practices and local dog management, but it reinforces the idea that transmission dynamics are context-dependent and cannot be inferred from macroclimatic suitability alone.

Regarding intrinsic host factors, the results are consistent with previous studies reporting no clear breed or sex pre-disposition beyond differences attributable to management practices, such as those observed between hunting or working dogs and companion animals (4, 5, 10, 12, 26–29). Age appeared to act as a cumulative risk factor, which is consistent with progressive exposure in endemic areas. It should also be considered that passive sampling is inherently biased toward animals whose owners seek veterinary care, which may lead to underestimation of the true burden of infection in stray dog populations or in neglected rural areas, where infection pressure is likely to be higher.

This study has several limitations. First, because samples were collected through veterinary clinics, the dataset primarily represents owned dogs, and the potential contribution of stray dogs as an uncontrolled reservoir could not be assessed. In addition, the selection bias inherent to clinical sampling means that the estimated regional risk may partly reflect differences in owners' healthcare-seeking behavior. The temporal increase observed in 2024, particularly in northern Spain, should therefore be interpreted with caution, as it may reflect not only favorable climatic conditions, including extreme heatwaves, but also increased surveillance and more proactive screening by veterinarians in emerging areas. Second, information on chemoprophylaxis was not available for the included dogs. In regions with greater access to veterinary care and higher compliance with preventive measures, the realized transmission risk may therefore appear lower than the underlying environmental suitability. Third, although the antigen tests used have very high specificity (100%), their imperfect sensitivity (94%) may have led to under-detection of some infections. In particular, infections with low female worm burdens, male-only infections, or pre-patent stages may not always be identified. Consequently, our findings should be interpreted as a conservative estimate of the geographic distribution and transmission risk intensity of *D. immitis* in the studied regions.

In conclusion, the multivariate analysis indicates that geographical region was the main determinant of RTR, with the archipelagos showing the highest risk values, followed by a clear expansion pattern across the Iberian Peninsula. The association of increased risk with indoor habitats and semi-arid climates broadens the conventional epidemiological profile of heartworm disease and suggests that urbanization and environmental modification may be as relevant as macroclimatic conditions in shaping transmission. From a One *Health* perspective, the high circulation of the parasite in dogs, together with the presence of vectors adapted to indoor and urban environments, may increase the risk not only of canine heartworm disease but also of human pulmonary dirofilariasis and other parasite-related hypersensitivity phenomena in exposed populations (30). These findings support the need for geographically tailored prevention and surveillance strategies, with particular emphasis on year-round prophylaxis in the islands and southern areas, and on active surveillance and risk awareness in inland regions of the peninsula.

## Data Availability

The original contributions presented in the study are included in the article/Supplementary material, further inquiries can be directed to the corresponding authors.
